# Low-density neutrophil subsets in allergic rhinitis: heterogeneity and an emerging hypothesis for the non-type 2 endotype

**DOI:** 10.3389/falgy.2026.1934796

**Published:** 2026-10-19

**Authors:** Pengyuan Yu, Feihu Wu, Yufeng Chen, Yun Li, Xinyue Zhang, Yan Ruan, Gang Liu, Xinyu Han

**Affiliations:** 1 Jiaxing Hospital of Traditional Chinese Medicine, Jiaxing, Zhejiang, China; 2 Shuguang Anhui Hospital Affiliated to Shanghai University of Traditional Chinese Medicine, Hefei, Anhui, China; 3 The First Affiliated Hospital of Anhui University of Chinese Medicine, Hefei, Anhui, China; 4 The First Affiliated Hospital of Guangzhou University of Chinese Medicine, Guangzhou, Guangdong, China

**Keywords:** allergic rhinitis, low-density neutrophils, LOX-1, neutrophil extracellular traps, neutrophil heterogeneity, non-type 2 inflammation

## Abstract

Allergic rhinitis (AR) is conventionally viewed through a type 2/eosinophil-centric lens, yet a substantial subgroup of patients exhibits a neutrophil-predominant, corticosteroid-refractory inflammatory phenotype that responds poorly to standard therapy. Once regarded as a homogeneous, terminally differentiated lineage, neutrophils are now recognized as a functionally heterogeneous population. Among their subsets, low-density neutrophils (LDNs)—encompassing pro-inflammatory low-density granulocytes and immunosuppressive polymorphonuclear myeloid-derived suppressor cells—have emerged as key regulators of innate and adaptive immunity. This Mini Review synthesizes current evidence and proposes an emerging hypothesis that LDN heterogeneity may contribute to, rather than define, the non-type 2 endotype of AR. We discuss (i) the identification and dual functionality of LDNs; (ii) AR-specific findings, including CD16/CD62L-defined neutrophil subsets and recently described circulating LOX-1 + neutrophils that activate T cells; (iii) the neutrophil-T cell crosstalk that may amplify non-type 2 inflammation; and (iv) the translational potential of LDNs as biomarkers and therapeutic targets. A tighter characterization of LDN subsets may enable endotype-guided, neutrophil-directed therapy in AR.

## 1 Introduction: beyond type 2—the neutrophil dimension in allergic rhinitis

Allergic rhinitis (AR) affects up to 40% of the population and is classically driven by IgE-mediated, type 2 inflammation dominated by eosinophils and Th2 cytokines (1, 2). However, this paradigm is incomplete. Nasal cytology routinely identifies a neutrophilic AR subgroup (AR(Neu)), in which local neutrophils predominate and which responds poorly to intranasal corticosteroids and antihistamines (2). Parallel observations in asthma estimate that 20-30% of patients display a neutrophil-predominant, type 2-low phenotype resistant to biologics (3). Neutrophils constitute 50-70% of circulating leukocytes and were long considered short-lived, antimicrobial effector cells; contemporary work reveals marked phenotypic and functional plasticity (4, 5).

In type 2-mediated disorders neutrophils were historically cast as bystanders, yet they are now recognized as active contributors: neutrophilic inflammation characterizes the type 2-low phenotype of asthma and chronic rhinosinusitis and predicts attenuated responses to type 2 biologics (3, 4). Whether neutrophils merely accompany or actively shape type 2 versus non-type 2 polarization is the central question addressed in this review. The recent recognition that neutrophils subdivide into subsets with distinct, sometimes opposing, immunomodulatory roles has reopened this question for AR (1, 5).

Neutrophil identity is not fixed; it is shaped by multiple determinants, including anatomical niche (blood versus tissue), the inflammatory stimulus and disease state, microbial exposure, metabolic status, sex, and developmental stage (5, 6). Age is one such axis: neonatal neutrophils display defective neutrophil extracellular trap (NET) formation and distinct functional programs compared with adult cells, underscoring the developmental plasticity of the compartment (6). This Mini Review focuses on one particularly instructive axis of neutrophil heterogeneity—the low-density neutrophil (LDN) compartment—and proposes the emerging hypothesis that LDN subset balance may contribute to the non-type 2, neutrophil-driven endotype of AR. Whether this linkage holds in human AR remains to be established.

## 2 Low-density neutrophils: identification and functional duality

Two classification schemes are frequently conflated in the neutrophil literature and are kept strictly distinct in this review. First, LDNs are an operational entity defined by physical density: cells recovered in the low-density (buoyant) fraction during density-gradient centrifugation, irrespective of surface phenotype (7, 8). Second, phenotypic neutrophil subsets—for example CD16hiCD62Ldim (activated) or LOX-1 + neutrophils—are defined by surface markers and may or may not co-purify with the low-density fraction. Whether CD16hiCD62Ldim or LOX-1 + neutrophils in AR are enriched within the low-density fraction is not established; the two terminologies are therefore not used interchangeably below.

Far from being a technical artifact, the buoyant LDN population is expanded across a wide range of inflammatory, autoimmune, infectious, neoplastic, and cardiovascular conditions and displays context-dependent functions (7–9). The LDG-versus-PMN-MDSC dichotomy is a useful simplification, but LDNs are in fact more heterogeneous: beyond these two poles they include tumor-associated PMN-MDSCs that enforce immune evasion in cancer, IBD-associated neutrophils, LDGs expanded in viral infection and COVID-19, and subsets described in vaccine responses and cardiovascular disease, each with distinct transcriptional and functional programs (7–9). Two functionally opposed poles are nevertheless repeatedly described. Low-density granulocytes (LDGs) are pro-inflammatory and are intrinsically primed for spontaneous NET release—a property that links them to tissue injury, thrombosis, and extracellular-trap-mediated inflammation (10–13). By contrast, polymorphonuclear myeloid-derived suppressor cells (PMN-MDSCs) are immunosuppressive, constraining T-cell proliferation through arginase-1, reactive oxygen species, and nitric oxide (7, 8, 14). Crucially, the same surface phenotype (e.g., CD11b + CD15 + CD33 + CD66b + CD14-, with variable LOX-1 expression) can map onto either behavior depending on disease context (11, 14). In systemic lupus erythematosus, for example, LDGs are not suppressive; instead they produce interferon-gamma and tumor necrosis factor-alpha and promote Th1 responses, behaving as pathogenic, non-suppressive cells despite expressing the canonical PMN-MDSC marker LOX-1 (10, 11). Thus, LDN ‘identity’ is not fixed but is decoded by anatomical niche and activation state (7, 8). This duality is central to understanding AR, where neutrophils may simultaneously dampen and exacerbate inflammation; the potential contribution of NETs released by LDNs to AR and related upper-airway allergy is considered below and summarized in Table 1 (15, 16).

**Table 1:** Low-density and phenotypically defined neutrophil subsets reported in allergic rhinitis and related upper-airway allergy.

Subset (marker definition)	Disease/condition	Sample size	Cell source & method	Key finding (markers)	Major limitation	Ref.
Activated/end-state neutrophil (CD16hiCD62Ldim; CD16dimCD62Ldim)	AR (pollen season)	AR *n* = 8, HC *n* = 6	Blood, nasal biopsy, nasal lavage; flow cytometry (CD16/CD62L)	CD16hiCD62Ldim predominates in nasal tissue, primes T cells & eosinophil migration; CD16dimCD62Ldim accumulates in lavage	Small sample; seasonal (pollen) cohort; no link to clinical endotype shown	(1)
LOX-1 + neutrophil (CD14-CD11b + CD15 + LOX-1+)	AR	Not reported	Peripheral blood; flow cytometry; CFSE T-proliferation; ELISA	Increased in AR; promotes CD3+ T-cell proliferation & cytokines; independent of IgE/age/sex	Sample size not reported; cross-sectional; direction of causality unproven	(7)
Density-converted neutrophils (low-density fractions)	Allergic subjects/asthma	Not reported	Density gradients (4 fractions: >1.085, 1.081-1.085, 1.077-1.081, <=1.077 g/mL); chemotaxis	Allergic subjects have greater proportion of lower-density fractions; PAF/GM-CSF convert density *in vitro*	Older study (1990); n undefined; non-standard density cutoffs	(8)
LDG, pro-inflammatory (CD11b + CD15 + CD66b + LOX-1+)	SLE (parallel model)	SLE *n* = 94, HC *n* = 80	Fresh blood; density LDG/NDG fractionation; flow; confocal	LDGs activate T cells, non-suppressive despite LOX-1; produce IFN-gamma/TNF-alpha	SLE model, not AR; extrapolated to AR by analogy	(10, 13)
PMN-MDSC, suppressive (CD11b + CD15 + CD14-CD66b+)	Human systemic inflammation/allergic asthma (mouse)	Human *n* = 7 (LPS challenge); mouse varies	Blood (Ficoll PBMC); flow CD16/CD62L; FACS; mouse OVA models	Suppressive subset inhibits T cells via Mac-1 (human); attenuates airway inflammation via ILC2 (mouse)	PMN-MDSC role in human AR not directly shown; mouse data extrapolated	(9, 14, 15)
NETs (CitH3/MPO/NE extracellular DNA)	Rhinitis (infectious vs allergic)	Not reported	Nasal mucus; SytoxGreen, microBCA, micrococcal nuclease, ELISA	NET structures confirmed in infectious rhinitis; in allergic rhinitis only small non-NET eDNA, no NET-DNA	NETs not observed in allergic cohort; AR relevance unconfirmed (exploratory)	(18)
Interferon-induced (ISGhi) neutrophils/NETs	AR (mouse model + human phase-2 RCT)	Mouse OVA/HDM models (*n* = 3-5/group); human RCT *n* = 42	Mouse nasal lavage/mucosa; flow cytometry (ISGhi neutrophils); metatranscriptomics; phase-2 RCT nasal lavage	Nasal commensal viruses -> type I IFN -> ISGhi neutrophils release NETs promoting AR; ribavirin/IFN-sensor blockade reduces NET activity & symptoms	Mouse + early human trial; human AR NET mechanism inferred; distinct viral/IFN axis from LDN hypothesis	(27)
NET-to-neutrophil ratio	CRSwNP	117 CRSwNP patients	Nasal polyp tissue; immunofluorescence (CitH3-NE); manual count	NET ratio predicts postoperative refractoriness in neutrophilic endotype	CRS, not AR; indirect inference for AR	(20)
NET-driven basal cell hyperplasia	CRS	45 sinonasal biopsies (CRSsNP 11, CRSwNP 9, eos NP 8, noneos NP 10, HC 7)	Tissue immunofluorescence; murine model	NETs promote *Δ*Np63 + basal cell hyperplasia	CRS model; DNase/sivelestat effects in AR inferred	(21)

AR, allergic rhinitis; CRS, chronic rhinosinusitis; LDG, low-density granulocyte; LDN, low-density neutrophil; NET, neutrophil extracellular trap; PMN-MDSC, polymorphonuclear myeloid-derived suppressor cell; SLE, systemic lupus erythematosus.

## 3 From phenotype to endotype: defining the neutrophilic (non-type 2) Ar endotype

Allergic rhinitis is defined as an IgE-mediated, symptomatic inflammation of the nasal mucosa induced by allergen exposure, clinically manifested by sneezing, rhinorrhea, nasal pruritus, and obstruction (1, 2). Although clinically unified, AR is not a single disease but a syndrome that can be decomposed into mechanistically distinct endotypes.

Two principal endotypes are recognized. The type 2 (eosinophilic) endotype—AR(Eos)—is driven by IgE, eosinophils, and Th2 cytokines and typically responds to intranasal corticosteroids and antihistamines (2). In contrast, the non-type 2 (neutrophilic) endotype—AR(Neu), in which nasal neutrophils predominate (>=90% of inflammatory cells)—is type 2-low and responds poorly to conventional therapy but may improve with macrolide immunomodulation (2, 17). Mixed and paucigranulocytic forms also exist (2). The defining biological characteristics that operationalize AR(Neu) are (i) neutrophilic nasal cytology separable from AR(Eos) and mixed forms; (ii) a type 2-low immunologic signature, with blunted IgE/eosinophil/Th2 axes and relative enrichment of type 1/Th17 and neutrophil-associated mediators; and (iii) a distinct therapeutic response—limited benefit from intranasal corticosteroids plus antihistamines but marked improvement when switched to macrolide immunomodulation (2, 17).

Crucially, a neutrophilic nasal cytology pattern is not, by itself, proof of a stable AR endotype. Nasal neutrophilia is a shared downstream response to several stimuli and must be interpreted in context. Recognized non-endotypic drivers include concurrent upper-respiratory infection (viral or bacterial), environmental irritants and air pollution, cigarette smoke, topical medication effects (e.g., prolonged intranasal decongestant or certain inhaled drugs), and comorbid chronic rhinosinusitis, which independently sustains neutrophil influx (18, 19). Non-allergic rhinitis with eosinophilia and other non-allergic subtypes further complicate the picture. Attribution of nasal neutrophilia to a primary AR endotype should therefore be made after excluding these confounding exposures, and longitudinal sampling is preferable to a single time point. The hypothesis developed in this review is that, once such confounders are accounted for, an LDN-enriched neutrophil compartment characterizes the genuine non-type 2 AR endotype.

## 4 Low-density neutrophils in allergic rhinitis: from Lox-1 + cells to Cd16/Cd62l subsets

Human AR studies establish that neutrophils in AR are phenotypically heterogeneous (1, 20, 21). By contrast, the functional assignment of these subsets—pro- versus anti-inflammatory—rests largely on parallel work in systemic lupus erythematosus, asthma, or mouse models; such findings are noted as illustrative and are not assumed to hold automatically in AR.

The first human clue that AR neutrophils are heterogeneous came from subclassification by CD16 (FcgammaRIII) and CD62L (L-selectin) expression. Arebro and colleagues showed that, during the pollen season, AR patients have elevated neutrophils in blood, nasal biopsies, and nasal lavage, and that the activated subset (CD16highCD62Ldim) predominates in nasal tissue whereas CD16dimCD62Ldim (‘end-state’) cells accumulate in lavage (1). These activated neutrophils primed T cells and enhanced eosinophil migration *in vitro*, supporting an active, immunomodulatory role rather than passive tissue infiltration (1). The three canonical subsets—segmented (CD16brightCD62Lbright), banded/immature (CD16dimCD62Lbright), and hypersegmented/suppressive (CD11cbrightCD62LdimCD16bright)—were formally defined in human systemic inflammation and provide the reference framework for AR studies (14), as summarized in Table 1.

Direct evidence of an activated, pro-inflammatory neutrophil subset in AR arrived with the characterization of circulating LOX-1 + neutrophils (CD14-CD11b + CD15 + LOX-1+). Deng and co-workers reported that LOX-1 + neutrophils are significantly increased in the peripheral blood of AR patients and, unlike classical PMN-MDSCs, promote CD3+ T-cell proliferation and pro-inflammatory cytokine release; importantly, their levels correlated with neither IgE, age, nor sex, suggesting independence from classical type 2 sensitization (20). This mirrors the systemic lupus erythematosus LDG phenotype, where LOX-1 + cells are pro-inflammatory rather than suppressive (10); the ‘PMN-MDSC marker’ LOX-1 in fact flags an activated, T-cell-stimulatory neutrophil in AR. Whether these LOX-1 + cells reside in the density-defined LDN fraction is, however, not yet demonstrated and should be tested directly.

The notion that allergy reshapes neutrophil density is not new. Already in 1990, Miyagawa and colleagues demonstrated that allergic subjects harbor a greater proportion of lower-density neutrophil fractions and that platelet-activating factor and granulocyte-macrophage colony-stimulating factor convert neutrophil density *in vitro*, implying that soluble mediators in the allergic milieu can generate LDN-like buoyant neutrophils (21). Whether NETs operate in the AR niche is, by contrast, unresolved. In a dedicated analysis of upper-airway secretions, NET structures—extracellular DNA fibers co-localizing with myeloperoxidase and neutrophil elastase—were clearly detected in infectious rhinitis but were not observed in allergic rhinitis, where only small amounts of non-NET extracellular nucleic acids were found (15). Extending beyond human secretions, a 2026 study in ovalbumin- and house-dust-mite-induced mouse AR models showed that interferon-induced (ISGhi) nasal neutrophils release NETs that promote AR, whereas clearing nasal commensal viruses (ribavirin) or blocking interferon sensors suppressed NET activity and alleviated disease (16). NET release by neutrophils is therefore established as a pathogenic mechanism in experimental AR but remains to be confirmed in human AR secretions, where its value as a biomarker or therapeutic target is currently exploratory (13, 15, 16).

## 5 Neutrophil-T cell crosstalk: a non-type 2 amplifier (candidate mechanism)

Whether LDNs are actually present and functionally consequential in human AR remains unproven; the neutrophil-T cell axis nevertheless offers a candidate mechanism by which a low-density, immunomodulatory neutrophil compartment could amplify non-type 2 inflammation. Several of the interactions below are documented outside AR (systemic inflammation, systemic lupus erythematosus, mouse models of asthma) and are presented as candidate mechanisms pending AR-specific confirmation. Activated CD16highCD62Ldim neutrophils from AR patients prime T cells and augment eosinophil recruitment (1), while LOX-1 + neutrophils directly drive T-cell proliferation and cytokine production (20). In systemic lupus erythematosus, LDGs similarly break T-cell anergy and favor Th1 responses (10). A regulatory counterweight exists: a CD11cbrightCD62LdimCD16bright neutrophil subset can suppress T-cell proliferation via Mac-1-dependent, hydrogen-peroxide-mediated mechanisms (14), and PMN-MDSCs attenuate allergic airway inflammation by restraining group 2 innate lymphoid cells in mouse models (22). More broadly, myeloid-derived suppressor cells are now recognized as pathologically activated neutrophils and monocytes with distinct genomic and metabolic programs that separate them from classical neutrophils (9), and in mouse models of asthma granulocytic MDSCs track with elevated Th17 cells (23). The net inflammatory output in AR may therefore reflect the balance between pro-inflammatory LDGs/LOX-1 + neutrophils and suppressive PMN-MDSCs (10, 20, 22). Because the pro-inflammatory LDN signals (T-cell activation, type 1/17 skewing) are independent of IgE and eosinophils, they provide a plausible mechanistic substrate for the non-type 2, neutrophil-rich AR endotype that escapes conventional type 2-directed therapy (2, 20)—a hypothesis that remains to be verified in human AR cohorts.

## 6 Low-density neutrophils as biomarkers and therapeutic targets in the non-type 2 endotype

Translating LDN biology into the clinic begins with endotype stratification. Nasal cytology already separates AR(Eos) from AR(Neu) (neutrophils >=90% of inflammatory cells); prospective data show that AR(Neu) patients gain limited relief from intranasal corticosteroids plus antihistamine, whereas those switched to clarithromycin—exploiting its anti-neutrophilic, immunomodulatory properties (17)—improve substantially (2). For biomarkers, nasal cytology (AR(Neu) vs AR(Eos)) and circulating LOX-1 + neutrophil counts are the best-supported candidate readouts in AR (2, 20); both require prospective validation. NET burden—where detectable—has been proposed as a secretion-based biomarker, but given that NETs have not been confirmed in allergic rhinitis secretions, this application is currently exploratory (13, 15). In the related upper-airway disease chronic rhinosinusitis with nasal polyps (CRSwNP), the NET-to-neutrophil ratio independently predicts postoperative refractoriness in the neutrophilic endotype (24), and DNase I or the neutrophil elastase inhibitor sivelestat reduces polyp formation by blocking NET-driven basal-cell hyperplasia (25); these findings derive from CRS and are cited to illustrate a therapeutic principle that may, but has not yet been shown to, transfer to neutrophil-dominated AR.

Translating LDN biology into therapy is most coherent when strategies are matched to the LDN compartment rather than to neutrophils in general. (i) Phenotype-selective targeting: anti-LOX-1 is the only approach directed at a marker enriched on the pro-inflammatory LDG/AR LOX-1 + neutrophil and could selectively blunt T-cell activation without broadly immunosuppressing (10, 20). (ii) Function-selective targeting: NET inhibition (e.g., DNase I) addresses the LDG-prone NET phenotype where NETs are operative—established in CRS (24, 25) and, in experimental AR, by interferon-induced neutrophils (16)—but it is unproven as an AR therapy. (iii) Compartment rebalancing: macrolide immunomodulation may shift the LDG/PMN-MDSC balance and is the best-supported intervention for AR(Neu) (2, 17), although its effect on LDN subsets is untested. (iv) Niche-targeted approaches: the nasal microbiome shapes the neutrophil-rich niche; nasal commensal Staphylococcus aureus can modulate IL-33-dependent type 2 responses in allergic nasal mucosa (26), and anti-staphylococcal strategies are a microbially informed avenue. (v) Recruitment blockade: pollen and cat-dander allergens instigate innate neutrophil influx through Toll-like receptor 4/myeloid differentiation protein-2/CXCR2 signaling, and pharmacological CXCR2 antagonism abrogates allergic sensitization and inflammation in mouse models (27). Note that IL-33 is a type 2 epithelial alarmin; no direct link to LDNs or the non-type 2 AR endotype has been established, so it is not presently an LDN-directed target (26). These strategies constitute a neutrophil- and LDN-directed portfolio for the non-type 2 AR endotype, but their efficacy in molecularly defined AR(Neu) patients is unproven and represents a key trial gap.

## 7 Conclusion and future perspectives

Low-density neutrophils may be a key, under-appreciated component of the non-type 2 AR endotype rather than a mere footnote to eosinophilic disease. The evidence reviewed here suggests that (i) AR neutrophils are functionally heterogeneous, with LOX-1+/LDG subsets promoting T-cell activation independently of IgE in related models (10, 20); (ii) the LDN compartment could bridge innate recruitment and adaptive amplification (1, 5, 20); and (iii) LDN-associated readouts (nasal cytology, LOX-1 + counts) are candidate biomarkers, whereas NET burden remains exploratory: NET release promotes experimental AR (16) yet NETs were absent from allergic rhinitis secretions (15), leaving human relevance unproven (2, 15, 16, 20). Key knowledge gaps remain. Standardized gating strategies for human LDN subsets are lacking, hindering cross-study comparison (7, 14); whether CD16hiCD62Ldim or LOX-1 + neutrophils co-purify with the low-density fraction in AR is untested; longitudinal cohorts are needed to determine whether LDN profiles predict disease chronicity or treatment response (2, 20); and interventional trials of neutrophil-directed agents (macrolides, DNase, CXCR2 inhibitors, anti-staphylococcal approaches) in molecularly defined AR(Neu) patients are essentially absent (17, 25–27). Resolving these gaps will require the field to treat neutrophils—not merely eosinophils—as candidate endotype determinants in AR (Figure 1).

**Figure 1:**
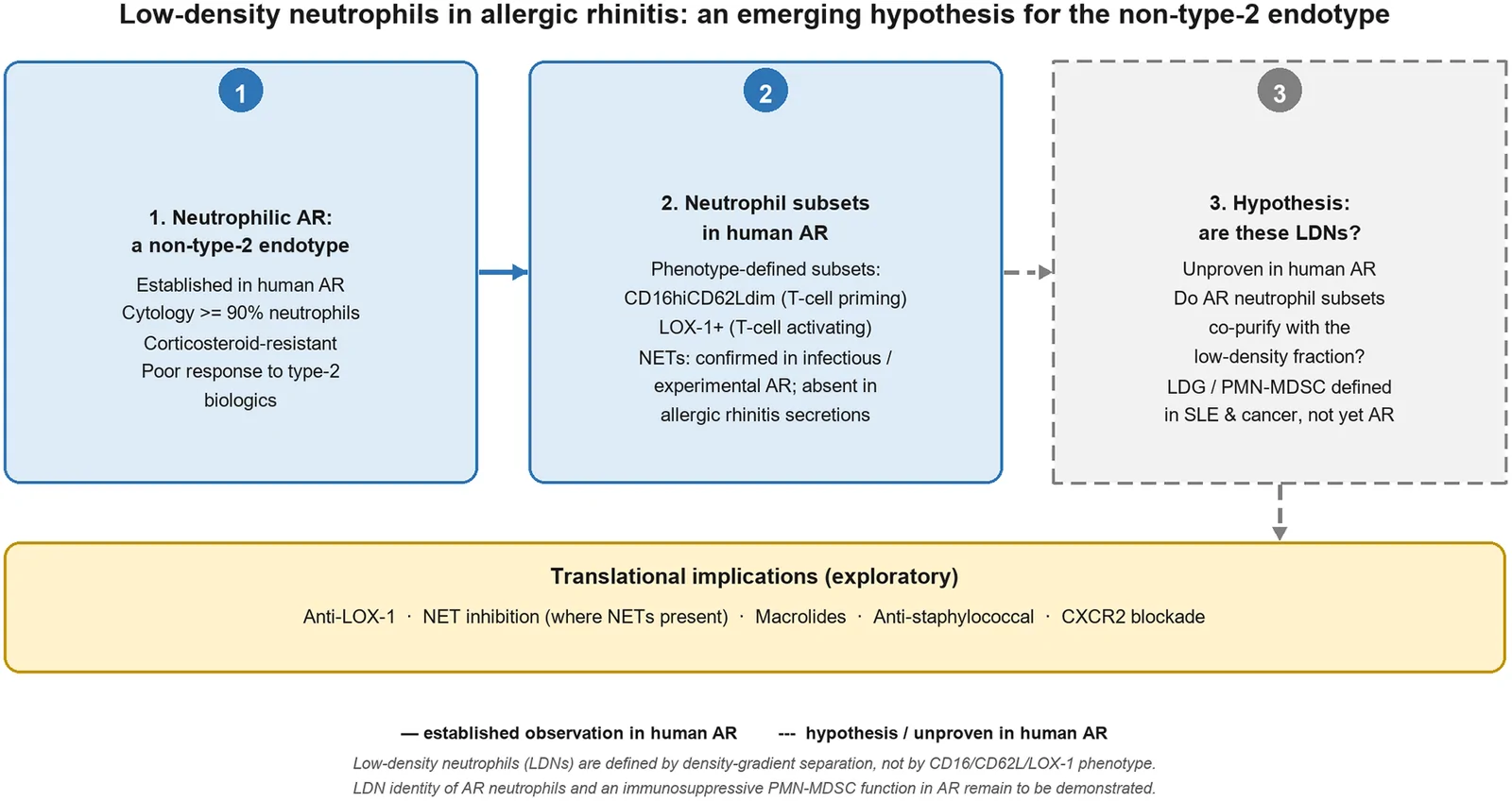
A three-step narrative for the proposed non-type-2 allergic rhinitis (AR) endotype. Step 1. Established in human AR: a neutrophilic AR(Neu) endotype defined by nasal cytology (>=90% neutrophils), corticosteroid resistance, and poor response to type-2 biologics. Step 2. Established in human AR: phenotype-defined neutrophil subsets—CD16hiCD62Ldim (T-cell priming) and LOX-1+ (T-cell activating) neutrophils; neutrophil extracellular traps (NETs) are confirmed in infectious and experimental AR but are absent from allergic rhinitis secretions. Step 3. Hypothesis (unproven in human AR): whether these AR neutrophil subsets co-purify with the density-defined low-density neutrophil (LDN) fraction—and thereby drive the non-type-2 endotype—remains to be demonstrated; low-density granulocytes (LDGs) and polymorphonuclear myeloid-derived suppressor cells (PMN-MDSCs) are well characterized in systemic lupus erythematosus and cancer but not yet in AR. Translational implications (anti-LOX-1, NET inhibition where NETs are present, macrolides, anti-staphylococcal, CXCR2 blockade) are exploratory. Solid elements denote established observations; the dashed box denotes a hypothesis.
